# Determinants of implementation of child-parent psychotherapy to treat early childhood trauma: a reflexive analysis

**DOI:** 10.1186/s12913-025-12937-w

**Published:** 2025-07-03

**Authors:** Silje Marie Haga, Heidi Jacobsen, Thomas Engell

**Affiliations:** Regional Centre for Child and Adolescent Mental Health, Eastern and Southern Norway, PO Box 4623, Nydalen, Oslo, N-0405 Norway

**Keywords:** Child-parent psychotherapy, Reflexive thematic analysis, Implementation, Trauma symptoms, Young children

## Abstract

**Background:**

Trauma experiences in early childhood can significantly impact children’s development. Child-Parent Psychotherapy (CPP) is an evidence-based treatment that addresses traumatic stress and child-parent attachment in children ages 0–6 years. Successful implementation of evidence-based interventions is challenging and presupposes a thorough understanding of the context in which it is being implemented. The objectives of this study were to explore the beliefs and experiences of therapists involved in the training and implementation of CPP in child and adolescent mental health- and child welfare services in Norway. The aim was to understand how health professionals experience implementation of CPP and identify determinants that could enhance or support the implementation process.

**Methods:**

Therapists were recruited from two subsequent CPP-training courses at the Regional Centre for Child and Adolescent Mental Health. We conducted ten semi-structured focus group interviews with forty therapists. They were interviewed after approximately six months of an 18-month CPP training initiative and again at the end of the training. We used reflexive thematic analysis to identify themes and determinants from descriptions of beliefs, attitudes and experiences with the training and implementation of CPP.

**Results:**

Four major themes were identified: (1) Intrinsic motivation due to alignment between personal needs and client needs, (2) Psychological readiness influences how therapists engage with CPP, (3) CPP offers welcomed flexibility and professional autonomy, but ambivalence emerges in practice, and (4) Leadership support is not the same as implementation leadership. These themes and determinants were often interconnected and multileveled, reflecting the complexity of implementation processes.

**Conclusions:**

Implementation of CCP was influenced by determinants such as therapists’ intrinsic motivation, psychological readiness, and a balance between flexibility and intervention structure. Proactive leadership or strong self-leadership appears to be necessary to create opportunities for implementation engagement. The successful implementation of CPP appears to require tailoring to individual, organizational, and cultural needs.

**Supplementary Information:**

The online version contains supplementary material available at 10.1186/s12913-025-12937-w.

## Introduction

Children from birth to the age of 5 are at particular risk of being exposed to potentially traumatic events [29]. Adverse experiences during early childhood can significantly impact the child’s neurological, cognitive, and emotional development [18, 20, 31, 41]. In meeting with these children and families, helpers may be uncertain and overwhelmed by the pain and chaos confronting them [25]. Therefore, a treatment that can help these children and families is most needed [43, 44].

Child-Parent Psychotherapy (CPP) is a relationship-based and trauma-informed treatment for preschool children who show symptoms of trauma and mental health challenges, have severe relationship difficulties, or are at risk of developing mental disorders based on traumatic experiences [28]. The treatment model is based on attachment theory and research and integrates modern psychodynamic, developmental, trauma, social learning, and cognitive behavioral theories and research [28]. The goal is to strengthen the relationship between the child and the caregiver, and to increasingly enable the caregiver to understand the child’s behavior, assist with emotional regulation, and support the child’s development [28]. CPP is primarily offered in child and adolescent mental health services, but also in child welfare services and other institutions where therapists can follow up with parents and children over time.

CPP assesses and addresses trauma in both caregivers and children, and the main part of therapy takes place with the child and caregiver together in the playroom [28]. In each therapy session, there is a vast array of possible topics and therapeutic pathways to choose from. Guided by what emerges moment by moment in the therapy room, the therapist chooses a port of entry and explores where it leads. In CPP, port of entry refers to the specific focus or starting point in therapy used to address the parent-child relationship or the child’s emotional needs. It typically involves identifying and addressing pivotal moments or dynamics—such as trauma, attachment disruptions, or emotional conflicts—to facilitate healing and strengthen their bond. The therapist also selects from various modalities, such as body language, dialogue, activities, or play [28]. The dyadic treatment is flexible, and the therapist continuously evaluates and adjusts goals and treatment focus. Fidelity to the treatment is not guided by a manual prescribing specific techniques but is instead rooted in overarching goals, principles, and potential intervention modalities, with a strong focus on reflective practice [28].

Efficacy studies of CPP have shown promising results for both children and caregivers [22, 24, 27, 30, 51]. While CPP is a well-established treatment method in the United States (US), the implementation of CPP in Norway is still in the early stages. Implementing evidence-based interventions in human services is considered complex because it often involves numerous dynamic elements, such as practices, processes, people, and systems [7, 15]. Such dynamics may be difficult to predict, standardize, and generalize across contexts. Implementation processes are stochastic at best, and successful implementation in one setting does not necessarily translate to another. Shared and unique barriers to implementation can be due to context, the implementation process, and intervention characteristics [16, 33], and successful implementation tends to emerge from how these elements interact and align. Consequently, transferring a US-based trauma-informed intervention to a Scandinavian service context may present new context-specific barriers due to societal, cultural, organizational, and individual differences [17]. Subsequently, efforts to address barriers and surface-level adaptations may be needed to achieve effective implementation that can be sustained over time [53, 55]. A recent study of 1287 implementations of evidence-informed interventions in the US found that 85% discontinued the intervention without ever achieving successful implementation [4]. The study found solid pre-implementation stages to predict implementation success and emphasized the critical importance of identifying and addressing context-specific barriers and facilitators to sustained implementation.

In a systematic review of determinants of implementing evidence-based trauma-focused interventions for children and youth, Powell et al. [45] identified a wide array of potentially important determinants at multiple levels and phases of implementation. They emphasized the importance of contextual understanding to inform ongoing implementation and adaptation. A recent study of determinants of implementation of CPP in Sweden [43] found determinants both overlapping with prior international studies (e.g., the importance of referral services, alignment with client needs, and time demands and workload) and unique ones to their context (e.g., challenges from concurring legal processes in the families). No prior study has explored determinants of implementation of CPP in a Norwegian context.

The present study explored the experiences and perspectives of health professionals involved in the training and implementation of CPP in child and adolescent mental health services in Norway. The aim was to understand how the health professionals experience implementation of CPP, identify key determinants that may foster or inhibit implementation, and solicit the therapists’ opinions on what might constitute appropriate remedial measures for future implementations.

## Methods

### CPP training and implementation

Two subsequent CPP trainings that lasted from 2017 to 2019 (cohort 1) and 2019–2021 (cohort 2) were the first efforts to implement CPP in Norway. CPP was implemented in 27 Norwegian services for children and families from 2017 to 2021, with implementation support from the Regional Centre for Child and Adolescent Mental Health in Norway, and the CPP developers. All participants voluntarily self-selected to partake in the CPP training and implementation, with permission from their service leaders. The CPP training included prework activities, three group training sessions over 18 months, biweekly zoom-based group reflective supervision and expert group consultation sessions, completion of CPP training cases, and technical assistance. The first training was facilitated by the international director of CPP dissemination and implementation, and the second training was facilitated by three Norwegian psychologists who were in the process of becoming trainers. The psychologists had regular meetings with the international director for supervision and guidance.

Core implementation strategies included educational material, a training program with ongoing group supervision (see below), audit and feedback, technical assistance, and formal commitment. Detailed reporting of implementation strategies cohorts is depicted in Table 1 in accordance with recommendations by Proctor et al. [46].

**Table 1:** Implementation strategies

Implementation strategy	Actor(s)	Actions	Action targets	Dose	Timing	Cohort 1	Cohort 2
Distribute educational material	Administrative staff, supervisor(s)	Distribute intervention manuals and fidelity forms to therapists.	-Provide therapists with the information and materials necessary to prepare for and complete CPP- training with fidelity. -Intended to increase clinicians’ capability and motivation to adhere to CPP.	311 page manual and a fidelity form with 24 fidelity items to score and rubrics for case descriptions and notes	The manual was distributed before training, and fidelity forms during the first training session.	X	X
Dynamic training	Cohort 1: Supervisor from the U.S (program developer, English-speaking) Cohort 2: Trained supervisors in Norway (Norwegian-speaking)	Plan for and conduct training in CPP in an ongoing way. Training includes didactic teaching, role-play, modeling, practice elements (e.g. ports of entry), and case review. In the later bulks of training, volunteers also provided a video recording of a session for the group to reflect on during training. All training was based on the CPP-manual and focused on the theoretical foundations of CPP, fidelity to CPP components, and principles of trauma-informed therapy with young children and caregivers.	- Develop therapists’ capabilities and motivations for using CPP with fidelity - Problem-solve emergent challenges and needs for adaptations - Self-reflection and feedback	The training was nine seven-hour days divided into three bulks. The first bulk was five days, and the second and third were two days. The supervised clinical work started after the first bulk. The training period lasted approximately 17 months total.	The first bulk occurred at the beginning of the training, the second after approx. 6 months, and the third after another 6 months.	X	X
Obtain formal commitment/Contracting	Service leaders, administrative staff	Require leadership to sign contracts per email stating their CPP priority and determination to implement it. Contracts included an agreement to allow the therapist to have 20% of their work hours devoted to CPP implementation and practice.	- Increase leadership commitment and priority - Ensure time allocation for CPP implementation and practice	One contract via email	Before training.	X	X
Supervision and case reflection with feedback	Supervisor(s) and therapists in training.	Supervise and support teams of therapists who are implementing the innovation and provide protected time to reflect on the implementation, share lessons learned, and support one another’s learning. A mandatory part of supervision is two case-review presentations per therapist, where they complete a case presentation template. The clinician should demonstrate appropriate skills in implementing all three phases of the intervention.	- Provide a space for case reflections, feedback, problem-solving, and collegial support. - Increase self-efficacy, self-reflection, motivation, and enhance fidelity.	Supervision is biweekly 60 min. digital meetings throughout the training-period, which lasts approx. 1,5 years.	Following the first training session, therapists meet digitally with the teacher(s) for supervision.	X	X
Technical assistance (TA)	Supervisor(s), administrative staff, therapists in training	TA was available at the therapists’ request. The most frequent TA activities included assistance with the use of digital tools (e.g., Zoom) and implementation resources (e.g., handouts for training and fidelity forms).	- Reassure therapists and help them focus on implementation - Reduce cognitive strain - Problem-solve emergent practical needs	As needed throughout the training.	Ongoing or when needed	X	X

### Participants

All 52 participants from both cohorts were invited, and 40 agreed to participate. Most participants were women (95%), and age ranged from 38 to 61 years, with an average of 49. The number of years being employed at the current workplace ranged from 1 to 20 years, with an average of 7.5 years. The study sample included psychologists, child welfare officers, social workers, nurses, and medical doctors from 27 Norwegian services providing interventions and support to children and families (17 child and adolescent mental health services, five child welfare services, three private practices, one from Alternative to Violence treatment center, and one from The Children’s House (“Statens Barnehus,” similar to children’s advocacy centers). The study was approved by the Norwegian Centre for Research Data (project nr.: 673042). Written consent was obtained before interviews.

### The study context

Services in the Norwegian health- and welfare sectors tend to have clear mandates, guidelines, and regulations provided by the state and municipal governments. However, they are granted a high degree of professional autonomy in exercising these directives through the interventions and support they offer to clients. The services also tend to practice highly trust-based leadership, and their work cultures are often influenced by ethical virtues of freedom, autonomy, and the uniqueness of individuals, while at the same time emphasizing the value of working evidence-based [16]. Reimbursements or other financial or structural incentives for specific interventions are rare, and services tend to vary substantially in the interventions, practices, and support they provide.

### Data collection

This qualitative study used semi-structured focus-group interviews. Therapists from two CPP-trainings (cohort 1 and 2) were included. Each CPP-training lasted 18 months. Six focus groups were completed approximately after 6 months (cohort 1: three focus groups, cohort 2: three focus groups) and four focus groups were completed towards the end of the training (cohort 1: three focus groups, cohort 2: one focus group). The number of participants in each focus group ranged from five to eight. Independent research assistants, with no prior knowledge of CPP and no relationship with the participants led the focus groups. It was made explicit that the interviewers were not involved in the CPP-training and research to encourage the participants to speak freely about the implementation of CPP.

Two interview guides were developed based on the research questions. The interview guide developed for the focus groups conducted after six months of implementation consisted of five main parts: (1) Learning the method, (2) Perceptions and (3) Relevance of CPP, (4) Barriers and (5) Facilitators to getting started (for full interview guide, see Appendix A). The interview guide developed for the focus groups conducted after the implementation period addressed (1) Acceptance and potential adaptations, (2) Feasibility, and (3) Sustainment (see Appendix B). Each focus group interview lasted approximately 60 min.

### Data analysis

This study used a phenomenological-hermeneutical approach [21], with reflexive thematic analysis (RTA) as the analysis method [6]. We chose this approach to explore the therapists’ subjective experiences, attitudes, and beliefs while at the same time acknowledging how we, as researchers, are influenced by our preconceptions, experiences, and interpretations in trying to understand the participants’ descriptions of their lived experiences.

### Researcher characteristics and reflexivity

The study authors have a background in psychology, with a particular focus on infant and child mental health and implementation of interventions. The first author attended the CPP training and numerous digital supervisions, which provided first-hand contextual insight regarding the processes and common themes that arose throughout the training. This insight might have made the author more prone to recognizing interview themes that resemble significant discussions during the training. The second author has extensive teaching and research experience within the area of child welfare services. In addition, the second author has experience with training and implementing the Attachment and Biobehavioral Catch-up Intervention (ABC) [12], which resembles CPP in some regard. The third author has expertise in implementation theory and science and extensive experience developing and implementing interventions for children and families. His interpretations of the data and results are likely influenced by his familiarity with common implementation barriers, facilitators, and mechanisms. Subsequently, he may be prone to make deductive rather than inductive interpretations. However, he did not participate in coding or initial theme identification, thus not influencing the inductive process of identifying main themes.

### Reflexive thematic analysis

The analysis followed the six steps for RTA [6]. Step 1; data familiarization and writing familiarization notes; 2) systematic data coding; 3) generating initial themes from coded and collated data; 4) developing and reviewing themes; 5) refining, defining, and naming themes; and 6) writing the report. While the six phases are organized in a logical sequential order, the analysis is not a linear process. Rather, the process is iterative, requiring the researcher to move back and forth through the phases as necessary [6]. The steps are detailed in Table 2.

**Table 2:** Data analysis procedure

Thematic analysis steps	Description
Step 1: familiarization with the data	The first and the second author familiarized themselves with the data by reading the transcripts several times and making notes of preliminary ideas. The first author also listened to the recordings. Subsequently, they read through the interviews again and wrote comments on the statements they thought might be relevant. During this phase, the authors did not have any immediate thoughts about what might be relevant so they wrote comments on most things that seemed like it might have a bearing on something.
Step 2: Systematic data coding/generating preliminary codes	During the coding process, the first author went through the entire data set and coded all transcripts successively and systematically. That is, all comments and reflections made by the participants that appeared relevant to the research question were coded in the following manner: when reading the data, an excerpt of data/text was given a code. Considerations were made throughout the dataset to determine whether new segments of data were captured with the existing codes, or whether a new code was needed. All data segments that were coded were cut and pasted into a new word-file resulting in a collated file with all the codes and associated texts.
Step 3: Generating initial themes from coded and collated data	The purpose of generating themes is to capture something important with regards to the research question. As a first step to explore patterns of meaning, similarly coded extracts, along with a description of each code, were placed together in a table. When reviewing the coded data, the authors noted a focus on benefits and barriers with regards to various topics and started generating initial themes from this perspective. For example, two themes: “CPP as a framework” and “Implementation leadership” were both referred to as either beneficial or a barrier. Upon reflection, however, the authors determined the need to move from a more summative report to a deeper interpretation of the data to address the research question more fully and be true to reflexive thematic analysis. Therefore, they reinterpreted the codes and met to discuss possible themes and reflect on differences in interpretation.
Step 4: Developing and reviewing themes	The first author presented a map of four overarching possible themes which were discussed with the second author. An inductive and interpretive approach in theme development was used.
Step 5: Refining, defining and naming themes	In line with reflexive thematic analysis, the first and second author reviewed all the data material again to better understand the central organizing idea and essence of the themes. The authors aimed to decide on a set of distinctive themes but also determine how the themes worked together to tell a story. Four distinct themes and determinants based on an interpretation of the codes were identified. Transcripts were reviewed to find suitable quotes to illustrate each theme and relevant names for the themes were decided.
Step 6: Interpretation of results and writing the manuscript	In addition to the first and second author, a third author with expertise in implementation science interpreted results considering implementation theory and evidence and iteratively discussed with the other authors before and during the writing of the manuscript.

### Coding

Coding of transcripts was done by cutting and pasting text into a word file where all the extracted data were coded and collated. A predominantly inductive coding approach was adopted to best represent meaning as communicated by the participants [6]. However, some degree of deductive analysis was employed to ensure that the open coding produced themes that were meaningful and relevant to the research questions.

We used both semantic and latent coding, which meant that information could be coded as the semantic meaning communicated by the respondent and the latent meaning interpreted by the researcher [42]. This approach reflects the combination of interpretive and constructive ontology and epistemology as underlying theoretical assumptions of the analysis. This combination of analytical paradigms affords due consideration to the meaning constructed and communicated by the participant and our interpretation of this meaning as researchers.

## Results

### Themes

Four overall themes were produced from analyses of the data. The themes were (1) Intrinsic motivation due to alignment between personal needs and client needs, (2) Psychological readiness influences how a therapist engages with a new treatment, (3) CPP is a framework that offers welcomed flexibility, but ambivalence emerges in practice, and (4) Leadership support is not the same as implementation leadership. These overarching themes captured several interconnected determinants of implementation, which will be described below. An overview of the themes and determinants are outlined in Table 3.

**Table 3:** Summary of main themes and determinants of implementation

Main themes	Implementation determinants
Intrinsic motivation due to alignment between personal needs and client needs.	• CPP addresses needs the therapists have personally experienced as professionals and individuals. • CPP is attractive to therapists because of specific characteristics of the method (sympathetic and helps the caregiver see through the lens of the child). • Learning CPP increases confidence as a professional.
Psychological readiness influences how therapists engage with CPP.	• Therapists embodying an “I can do this -mentality” are proactive early adopters. • Previous relevant experience can ease the initial implementation phase. • Encouragement and modeling from trainers and colleagues appear necessary for therapists who are less psychologically ready.
CPP offers welcomed flexibility and professional autonomy, but ambivalence emerges in practice.	• The flexibility is welcomed and appreciated, but the lack of structure and instructions poses challenges in practice. • Less experienced or less “ready” practitioners may benefit from more manualized structures until they reach proficiency in CPP. • A sufficient theoretical foundation is necessary to proficiently utilize the flexibility in CPP.
Leadership support is not the same as implementation leadership.	• Leadership support was present but insufficient because the support was most often passive. • The burden of creating space for training and implementation largely falls on therapists. • Experienced therapists created the necessary conditions for implementation by exercising effective self-leadership. • Proactive implementation leadership, or effective self-leadership, is needed for the implementation of CPP to be feasible.

#### Intrinsic motivation due to alignment between personal needs and client needs

All the therapists pointed out that CPP fills a gap in mental health services for children and families, and several also expressed how CPP addresses needs they have personally experienced as professionals and individuals. They emphasized how they have lacked a method for treating the youngest children who have experienced violence and other traumatic events.

It is very positive that CPP clarifies that babies and toddlers are also traumatized. Because it is often the case that the older children are sent for treatment, but the baby who has been lying there listening, they are often overlooked. And to sort of highlight that, no, they are actually also traumatized, and they also need treatment, and we have someone who knows something. (P1, cohort 1)

*For a long time*,* I have been thinking that I would like to work more with parents and children together*,* so when this method was offered*,* it was very suitable for me. (P2*,* cohort 2)*

They described how the existing treatment methods thus far have usually been aimed at either the parents or the child, while working with children and parents together is often necessary for trauma and major psychological stress.

I have missed being able to work with the child and caregiver together in the room. This training has given me the tools on how to do that. (P3, cohort 2)

*It has been repeated here that it is not the child’s symptoms that should be reduced*,* it is the relationship with the caregiver that should be able to carry it. It makes sense*,* we know how much a child can be healed in the relationship*,* so that is what we should help strengthen. I like that very much. (P4*,* cohort 2)*

The therapists described CPP as a very sympathetic method because it focuses on “what has happened to you” and not “what’s wrong with you.”

*In relation to what I do in the child welfare service*,* the perspective “what has happened to you*,* not what is the matter with you”*,* for me that very much pervades the CPP method*,* and it is a complete match for me! (P18*,* cohort 1)*

Another aspect the therapists appreciated was the focus on helping the caregiver see the world through the eyes of the child.

Part of the overarching thing is that meaning should be created for the child and the parent. And I think that it is so fundamentally vital in a way, in children’s lives. (P5, cohort 1)

*It is a very meaningful way of working with families*,* both because it is evidence-based and it is a holistic way of thinking about therapy that I like very much*,* that I can relate to. (P6*,* cohort 2)*

During the training, motivation was upheld, and support from trainers played an important role, as expressed by the therapists below when they were asked about the training.

*It has been very exciting. I just feel like “oh*,* I want to be good at this!” I feel like I’m absorbing knowledge*,* and I think the instructors are really good at giving us tools and important input on how we should work on these cases. (P4*,* cohort 2)*

The therapists also expressed how learning CPP made them feel more confident as professionals working with the child, the caregiver, and the surrounding system.

I think it’s great, very inspiring and I think it’s given me a huge boost as a therapist. Working with the relationship in the room, it is so incredibly exciting. (P19, cohort 1)

*If I hadn’t had CPP*,* I wouldn’t have had anything to offer where the child could be involved. Then you would just talk about something*,* instead of being in the room and discovering it together. Both discover the challenges and discover the solutions together. So*,* I think it’s a unique tool that I’m incredibly grateful to have. (P7*,* cohort 1)*

A particularly favored practice element in CPP was the systematic assessment of the caregiver’s own trauma history to help the therapist understand the caregiver’s previous and current behaviors, reactions, and capacity.

This is one of the few methods that inherently includes talking to parents about their own traumas. This structuring towards the parents is a very important supplement. (P4, cohort 2)

We work just as much with the parent as the child. That is the strength of the method. (P9, cohort 2)

The deliberate recognition of the caregiver’s life experiences seemed to make the therapists feel more empathic toward the caregiver.

*It was quite overwhelming for me*,* this story. I knew there was something there*,* but that it was so fierce. So*,* we must create the story for the child*,* and then we’ll see how much she (*referring to the caregiver*) manages. I would like to suggest a home visit to see if that can reduce the tension a bit. Because she is terribly afraid that she is not good enough. (P8*,* cohort 2)*

All the therapists had volunteered to attend the CPP-training, and some had downplayed the capacity demands of the training in order to get permission from their leader. In other words, they expressed a high level of intrinsic motivation.

*I’m afraid that if you are upfront with your employer*,* then many of them will say: you can just forget about it. Many of us who do this training*,* I mean we are above average dedicated. (P10*,* Cohort 1)*

#### Psychological readiness influences how therapists engage with CPP

Several contextual elements seemed to influence the therapists’ sense of being capable and ready to practice the therapeutic elements and principles in CPP. As illustrated by the quotes below, these aspects included personal attributes, past experiences, tolerance for engaging in real-time learning, encouragement and modeling from trainers, and support from leaders and colleagues.

Some of the more “ready” therapists preferred to just start practicing and learning through practical experience.

I feel that we learn as we go. At least for my part, I’ll have to start seeing if I can get it done, or I’ll probably get it done. But it will be challenging. (P11, cohort 2)

*It is a method that even if we had gone through all of it in the first week*,* we would not have learned it. It’s learning by doing. I just have to practice*,* to be in the room and bring the knowledge with me and then return to supervision to gain even more knowledge and understanding. We have to practice standing in those processes. (P4*,* cohort 2)*

Others needed more time to feel capable and in control before they engaged in practice.

It was difficult to get started. I almost had to start without knowing if I started correctly, if not, I don’t think I’d be able to start. Because first I was like waiting to feel that I knew the method. (P12, cohort 1)

*I’m so used to having plenty of time and watching things on film and being able to go back*,* but now I’m supposed to be wiser in the moment and understand what’s going on. (laughs). (P13*,* cohort 1)*

The therapists also described how having previous experience with working with children and caregivers can ease the initial phase of implementing CPP.

*I think that if you have experience of working with parents and children*,* then you are used to having to adapt a bit then and there. At least for me that is an advantage. It takes a lot to catch me off guard. In CPP*,* anything can happen. Sometimes you have kids who are kind of all over you*,* and you have to deal with it. The parents may sit and say nothing. Sometimes it can get a bit violent. And I think that if I wasn’t used to working with parents and children*,* I would probably have become quite insecure. (P14*,* cohort 1)*

Also, the therapists valued the trainer who “lowered the bar” in the beginning and emphasized that mastering CPP will take time:

It’s great to be reminded that it’s perfectly okay that you’re in a process, you’re in a learning process and there is room for that. (P12, cohort 1)

*I am so glad they said that in the beginning we will be doing therapy that is CPP’ish. (P15*,* cohort 2)*

Modeling from trainers also encouraged therapists to engage in practicing CPP, even if they did not feel completely comfortable with it yet.

*They are very committed supervisors. They are so keen and put themselves out there. It’s really great. It’s a bit like observational learning. I’ll give them that. (P9*,* cohort 2)*

Some therapists expressed how the highly experienced trainer sparked both enthusiasm and admiration, but for others, she raised the bar so high that starting to practice felt intimidating.

*I think that her (the trainer) input is much more interesting than mine. She is a very good therapist; she has lots of good reflections. So*,* I think it is more interesting to listen to her*,* than to get some half-baked comments in English from me. (P16*,* cohort 1)*

However, watching other therapists’ video recordings during supervision regulated feelings of being overwhelmed.

Coming here and watching video clips, and seeing how you guys do it, it was infinitely calming (laughs) because then we got to see a bit of practice, it was very ok. (P10, cohort 1)

*I think the supervision group is supportive*,* open*,* and those who have shared their cases have done a wonderful job presenting. Both the things they have mastered well*,* and the things they find difficult. I think that’s how we learn. Great! (P17*,* cohort 2)*

#### CPP offers welcomed flexibility and professional autonomy, but ambivalence emerges in practice

The therapists described CPP as a comprehensive framework where good, clinical work is put into a system.

You have to do a thorough assessment. Which is very appealing. And then it’s quite open how you work, and I’m also very keen on that, because you can’t follow a recipe, you have to be present there and then. (P5, cohort 1)

*CPP has a framework that we must use all the time*,* relate to. But it’s not that rigid*,* and I really like that. (P11*,* cohort 2)*

They described CPP as an open framework, and explained how the openness ensures there is nothing inherently negative about the method itself:

*CPP is so open. It’s basically clinical judgement. It is a framework in a way*,* a way of thinking. There is nothing in the method itself which we cannot think is good. After all*,* the focus is on the child’s best interests. So*,* I can’t see that there is anything negative with CPP. (P3*,* cohort 2)*

Flexibility was described as an advantage, and something therapists highly appreciated. They valued how they did not need to follow a manual strictly but could rather use their own clinical experience together with what emerged during the therapy sessions.

Yes, I think the fact that the method is so flexible is absolutely fantastic. And challenging, of course, there is a bit of tension in that, as has been talked about here, and I definitely don’t know the method well enough yet. (P18, cohort 1)

*And then you have overarching goals with what you do*,* but you have to use yourself and your own experiences and what comes up. (P5*,* cohort 1)*

When using CPP in practice, however, an ambivalence emerged. Some therapists expressed that they need and appreciate flexibility but also described that the lack of clear instructions was a challenge.

*I feel ambivalent. Because it’s both attractive and… a bit unsure of what I think about it*,* especially as it’s sort of… infinite port of entry… and that in that sense*,* I could probably wish for a bit more structure. At the same time*,* that’s part of what I like about it too*,* that it’s clinical… that is*,* in a way you make a clinical assessment*,* should I go in here or should I go in here. so… I don’t know if I should say that I’m for or against it*,* I just feel it as an ambivalence from time to time. (P5*,* cohort 1)*

The therapists expressed an essential need for a sufficient theoretical foundation when embarking on CPP. They pointed out that one needs knowledge about the influence of trauma on child development and parent-and-child relationships. Without it, the method is viewed as too unstructured and unclear:

*I think that the method is kind of too unclear if you don’t have trauma knowledge*,* if you don’t have developmental psychology*,* if you’re going to start a bit like that from scratch*,* then I think it’s going to be an unclear method. (P16*,* cohort 1)*

#### Leadership support is not the same as implementation leadership

The therapists experienced that their leaders supported their involvement in CPP training and implementation. However, they expressed that their leaders had a limited understanding of CPP as an intervention and what is required in order to learn and implement it. The leadership support appeared to be present but passive.

*The responsibility lies with us; it will not be facilitated in any way. The management is willing and ready for us to do the training. But beyond that*,* it is our task to create space for it. We don’t get any fewer cases; it’s not organized that way. (P3*,* cohort 2)*

Although attitudinal support for implementation was present, more proactive support and involvement from leaders appeared to be lacking. The therapists voiced frustration over the limited understanding of the time and capacity requirements imposed by the implementation, and the lack of help from leaders to facilitate day-to-day work conditions that enabled dedicated engagement with training and implementation. The burden of creating space for training and implementation largely fell on therapists, without them realistically having the opportunity to do so without going beyond their work hours.

*I don’t quite get it. Our service manager wants us to do this training*,* to learn yet another treatment method. I do think it is important for our clinic to be able to offer this treatment. BUT we must use our spare time to read and study. So*,* it doesn’t add up. (P4*,* cohort 2)*

However, some of the therapists expressed that even though their leader did not actively facilitate the work conditions they needed, they did so themselves by making priorities and restructuring their week.

*We help ourselves! There is no “you can set aside 20%”. It is not easy to put into practice. We have set up days when we will only work with CPP. It’s not like we’ve gone to the manager*,* and it’s not like the manager is going “how’s it going? Do you get to work with CPP?” I feel that we ourselves must take that responsibility. (P4*,* cohort 2)*

Nevertheless, the therapists generally expressed barriers in the inner and outer setting, such as competing priorities, time constraints, high caseloads, and other concurrent change processes, which require proactive implementation leadership for the implementation of CPP to be feasible.

But then there is the challenge, of course, in the system where I work, this training comes in addition to everything else. Even if my employer supports CPP, there is still an expectation that I should maintain all the usual workload, all my other cases, so it is a challenge then, to make sure that CPP doesn’t slip. (P14, cohort 1)

*If I were to do everything during work hours*,* I wouldn’t have had a chance… if we are to read the literature and go all in*,* in the way it is expected*,* then it requires quite a lot. So*,* it is important that the motivation is very much there. (P10*,* cohort 1)*

## Discussion

CPP is an evidence-based treatment of trauma in young children and their parents. Implementation of CPP has been widely studied in the US, but rarely in Europe. This qualitative study explored therapists’ experiences with implementation of CPP in Norwegian health and welfare services, and factors influencing the implementation of CPP (i.e., implementation determinants).

Through reflexive thematic analysis, we decided on four main themes capturing important determinants of CPP implementation: (1) *Intrinsic motivation due to alignment between personal needs and client needs*, (2) *Psychological readiness influences how therapists engage with CPP*, (3) *CPP offers welcomed flexibility and professional autonomy*,* but ambivalence emerges in practice*, and (4) *Implementation support is not the same as implementation leadership*. In line with prior studies of implementation of trauma-focused interventions [43, 45], and implementation frameworks such as the “Consolidated Framework for Implementation Research” (CFIR) [8] and “Exploration, Preparation, Implementation, Sustainment” (EPIS) [2], our themes and their determinants span the inner and outer context of implementation and characteristics, the implementation process, and the intervention itself. Both within and across these themes, important determinants appeared to be interconnected and multi-leveled – reflecting the complex nature of implementation processes.

The first theme centers around intrinsic motivation. A sense of purpose and meaningfulness seemed to be at the core of the therapists’ motivation. The therapists described how there has been a lack of evidence-based trauma treatment methods for the youngest children, and many families have been difficult to help because the difficulties are complex and there has been a lack of systematic methodology. The therapists seem to appreciate how CPP approaches the child in a compassionate way, focusing on “what has happened to you” rather than “what’s wrong with you”. Another aspect of CPP that the therapists appreciate and find meaningful is the opportunity to work with both the caregiver and child at the same time and ultimately be a conduit between the two. This way of working with families aligns with the therapists’ values and makes them highly motivated and committed to implementing CPP. The importance of personal values in implementation efforts is well recognized in prominent implementation theories such as the CFIR [8]. Organizational theories highlight alignment between innovations and staff characteristics as key to innovation adoption and sustainment [13]. In line with behavior change theories such as COM-B [38], alignment with values may positively influence the rational reflexive motivation that affects how the practitioners think and feel about CPP and the work it entails – they identify with it. A final aspect that seemed to motivate the therapists was the clients’ positive feedback and the feeling of increased confidence as a therapist. These findings align with self-determination theory (SDT), which proposes that a sense of autonomy, relatedness, and competence will make people feel motivated, empowered, and invested in their work, including when they meet obstacles [10]. Future studies can consider testing how alignment between intervention characteristics and personal values affects intrinsic motivation and, subsequently, implementation outcomes.

The second theme sheds light on how proactive implementation of CPP seems to also depend on what we term “psychological readiness”. In this context, we can define psychological readiness as therapists responding to the thought of engaging in CPP with a degree of positive affect and cognition sufficient to engage proactively. The feelings and thoughts they express resemble a combination of psychological safety and self-efficacy, which seems to instill an “I can do this-mentality” and prevent negative affect and insecurities that would reduce engagement. An interplay between individual characteristics, previous experience, and social support appears to strongly influence a therapist’s psychological readiness, which aligns with findings from a recent study on the implementation and sustainability of CPP in Sweden [43]. Our findings add that encouragement and modeling from the trainer can be particularly important, particularly for the ones who feel less psychologically ready to engage in new therapeutic skills. Hence, a crucial role of the trainer can be to offer the right amount of emotional support during the initial stages of implementation and encourage people to persist in the new behavior despite their initial lack of proficiency [19, 40, 52]. These findings suggest that a helpful pre-implementation process might include considerations of the staff’s psychological readiness and tailoring implementation strategies accordingly. For instance, therapists with lower levels of psychological readiness may require more proactive social support and supervision.

The third theme describes how the flexibility of the intervention was considered a significant asset but also a challenge for some. CPP is a complex method that does not abide by a manual in the traditional sense but rather integrates principles from a wide range of theories, including developmental psychology, attachment, and trauma theory. The freedom and opportunities that come with a principle-driven framework put much responsibility on the therapist, and some therapists found it difficult to begin and navigate without a more supportive structure to follow.

The therapists who expressed being comfortable with proactively creating structure themselves had extensive relevant experience or experienced receiving sufficient support and reassurance. Our results are consistent with a prior study exploring intervention characteristics in similar Norwegian services [16]. Engell et al. [16] found that highly experienced child welfare practitioners prefer and thrive with flexible interventions, while less experienced practitioners may need and prefer more manualized structures until they reach proficiency in delivering the intervention. Our findings add to these results by indicating that social support and coaching may compensate, which aligns with how self-determination theory suggests continuous social support can foster people’s tendency to be proactive rather than passive [10].

The ambivalence therapists expressed regarding the flexibility of CPP may also result from dissonance between what practitioners rationally value when they reflect on their practice, and the bounded rationality they face in their busy day-to-day decision-making [48]. Although practitioners may aspire to integrate CPP into their autonomous clinical practice, doing so can require a training and habituation period with a degree of frequent rational decision-making that significantly increases cognitive demands. The severity often involved in trauma cases can also add to those demands. As such, a more manualized structure, at least as an implementation strategy during a learning period, may provide choice architecture that eases the cognitive strain and enables more autonomous learning and habituation – like a structure to lean on while learning. Alternatively, and in line with behavioral economics approaches to implementation [5], strategic nudges (i.e., a gentle intervention or environmental cue that steers individuals towards desired behaviors) could be designed to support decision-making at points of CPP that practitioners find particularly challenging.

Finally, the fourth theme addresses the need for proactive support and involvement from leaders in facilitating conditions necessary for successful implementation. The attitudinal support, encouragement, and permission the therapists receive from their leaders appear to be necessary conditions for the implementation of CPP. However, this form of support appears to be insufficient because, for most practitioners, implementation and learning require adjustments in the workplace, such as time allocated to implementation activities and temporarily reduced caseload or down prioritization of other tasks. These findings align with theory and evidence highlighting proactive leadership behaviors as core elements of effective implementation leadership [1, 36, 54]. Proactive leadership behaviors such as strategic prioritization, time allocation, and active engagement in CPP appear to be needed unless therapists go “above and beyond” what can be expected of them [14]. Similar findings were reported by Pernebo et al. [43], where practitioners expressed a sense of loneliness in the absence of active support from leaders, and caseloads were rarely adjusted to accommodate implementation needs.

The need for leaders to proactively facilitate conditions for implementation also aligns well with COM-B theory, emphasizing the interconnectedness between capabilities, opportunities, and motivations to implement changes. The practitioners reported feeling ready and motivated for change. However, they could have benefitted from more physical and cognitive opportunities to do so (e.g., time and mental capacity). The “awkward” phase of practicing new skills tends to require more mental capacity, and temporarily creating space for developing proficiency may be necessary.

A few (of the more experienced) practitioners spoke confidently about how they autonomously created space for implementation without proactive leadership support. They did what was necessary because they perceived the benefits of CPP as worth it. Interpreting those perceptions and actions, they appear to exercise effective self-leadership [35] by (1) focusing their attention on the rewards implementation of CPP can provide rather than on limited physical opportunities for implementation, and (2) identifying tasks such as prioritization, setting boundaries, and effective time-management as part of their responsibility and skill set as professionals. In doing so, and in line with elements of self-leadership theory, they may be practicing a mindset that helps them overcome barriers and, at the same time, reinforce self-efficacy and self-determination, which may subsequently reinforce intrinsic motivation for implementation and overcoming barriers [23] – a cyclical mechanism reinforcing implementation. As such, self-leadership, a relatively unexplored construct in implementation science, may have valuable implications for individual-level implementation strategies and mechanisms. Strong self-leadership may also be particularly culturally relevant in Scandinavian countries, where leaders and employees highly value individual autonomy and trust-based leadership [11, 49]. Highly proactive implementation leadership may be unnatural or unaligned with the philosophy of some leaders, and some employees may interpret highly proactive implementation leadership as intrusive or micro-management. Strengthening self-leadership may help find an appropriate balance. Future experimental studies can consider testing whether, how, and for whom strengthening self-leadership affects implementation outcomes, and how that compares to strengthening formal implementation leadership.

The therapists described how they had to spend more time on their “CPP-cases” than other cases and typically had to use their spare time preparing for and processing cases. They also mentioned having to down-prioritize other tasks. These are typical examples of unintended consequences that can occur when introducing a new intervention or policy, sometimes referred to as ripple effects in the intervention and implementation literature [47] and system responses or dynamics in systems science [32]. These effects can extend beyond the immediate scope of the implementation effort and influence various aspects of the organization, individuals, and the systems in which they operate. The extent to which a leader understands and anticipates ripple effects and system dynamics can be crucial for implementation sustainment, as it can enable improved planning, resource allocation, and management of emerging challenges [32, 39, 50]. Future implementations of CPP may benefit from efforts to anticipate system dynamics and prevent unintended consequences.

Characteristics of the intervention itself remain less studied and understood as determinants of implementation processes compared to other determinants [26]. However, recent years have seen increased attention to how characteristics of the intervention, and how those characteristics align with context, influence implementation and sustainment [16, 34]. The consistent emphasis from participants on the positive characteristics of CPP as an intervention echoes a recent study investigating predictors of practitioners’ intentions to use evidence-based practices. In testing how organizational, individual, and intervention characteristics influence intentions to use interventions, Ahuna et al. [3] found that intervention characteristics, such as perceived value and usability, accounted for more than three times the variability in intentions to implement. That study also found that practitioners working with more experienced supervisors had stronger intentions to use, which our results also support. However, motivations and strong intentions are not necessarily sufficient for behavior change and sustained implementation over time. The current study implicates that structural and organizational characteristics and leadership behaviors may inhibit or facilitate implementation and sustainment of CPP, and that different traits in practitioners and context dependencies may call for differentiation in intervention structure and implementation strategies.

To summarize, the implementation determinants identified as important in the current study fit well within determinant implementation frameworks such as the updated Consolidated Framework for Implementation Research (CFIR 2.0, [9]). CFIR captures the complex, multi-level nature of implementation and echoes how CPP implementation was influenced by the characteristics of the intervention (e.g., its evidence-base, theoretical foundation, and complexity), the individuals involved (e.g., implementation leadership, provider values, self-efficacy, capabilities, opportunities, and motivation for implementation), the inner context (e.g., structural characteristics, culture, and relative priority), the outer context (e.g., client needs), and the process of implementing CPP (e.g., coaching and engaging providers). The alignment between influential factors identified in our inductive analysis and specific CFIR determinants speaks to the relevance and applicability of CFIR as a determinant framework. We also acknowledge that our results align with determinants in other prominent implementation frameworks, such as, for instance, EPIS [2] and the theoretical domains framework [37].

## Strengths and limitations

The present study has strengths and limitations. Interviews were conducted both early in the training and towards the end, which offered valuable insight into different parts of the implementation process. The sample size was large, and the rich dataset provided a variety of reflections about CPP. However, we cannot generalize the results from this one qualitative study, nor make inferences about causality. Not all therapists accepted the invitation to participate in the study, so there is a risk that the sample was skewed (e.g., the more optimistic participants agreeing). The first author took part in some of the training and may have been influenced by her experiences in coding and interpretations. However, the second author, the other coder, did not participate in any implementation activities. We did not execute member checking, so the respondents have not validated our results and interpretations. Due to the pandemic, the therapists had limited capacity to partake in the study towards the end, and we could only complete one focus group interview at follow-up for the second cohort. Finally, the study was conducted in Norway, and the findings may not be transferable to other settings.

## Conclusions

The implementation of CPP in Norwegian health and welfare services can depend on several interconnected determinants. This study suggests that therapists’ intrinsic motivation, psychological readiness, and the need for a balance between flexibility and structure in the intervention can be particularly influential. Also, both proactive implementation leadership and strong self-leadership among therapists may help overcome implementation challenges. Strong self-leadership may be especially relevant in Scandinavian contexts favoring trust-based leadership and high levels of professional autonomy. These findings highlight the complexity of implementation and the importance of tailoring strategies to both individual, organizational, and cultural needs.

## Supplementary Information

Supplementary Material 1.

Supplementary Material 2.

## Data Availability

The data that support the findings of this study are not openly available due to reasons of sensitivity and are available from the corresponding author upon reasonable request. Data are located in controlled access data storage at The Regional Centre for Child and Adolescent Mental Health, Eastern and Southern, Oslo, Norway.

## Abbreviations

CPP: Child Parent Psychotherapy
RTA: Reflexive Thematic Analysis
ABC: Attachment and Biobehavioral Catch-up Intervention
US: United States
CFIR: Consolidated Framework for Implementation Research
EPIS: Exploration, Preparation, Implementation, Sustainment
COM-B theory: Capability, opportunity, motivation, behavior change theory
