# Supplementary material for: Determinants of implementation of child-parent psychotherapy to treat early childhood trauma: a reflexive analysis
Source: BMC Health Serv Res. 2025 Jul 3;25:907. doi: 10.1186/s12913-025-12937-w (PMC12224486; doi:10.1186/s12913-025-12937-w)
Supplement: Supplementary file 1 — Supplementary Material 1. [file 12913_2025_12937_MOESM1_ESM.docx]

**Appendix A**

**Interview guide**

**LEARNING THE METHOD**

- How has it been learning the method?
  - Have you gained sufficient knowledge about the method?
  - Did you feel prepared to start using the method?
  - How have the guidance/consultations with Chandra been?
  - Is there anything that has been particularly good or bad about the training?

**PERCEPTIONS OF CPP**

- What do you think about CPP?
- Is there anything about CPP that you feel skeptical about?
- What is particularly positive about CPP?
- Are there any elements/aspects of CPP that you consider unnecessary?

**RELEVANCE OF CPP**

- For those of you who have started using CPP treatment, do you feel that CPP meets the needs of children and parents exposed to violence or trauma?
- How does this way of working function in practice?
- What works less well?
- How do parents and children respond to the method?

**BARRIERS TO GETTING STARTED**

- What are the obstacles to starting CPP treatment (why haven't you begun using CPP treatment)?
- Uncertainty about how to work with the method
- Have not had violence-exposed/trauma-exposed dyads for treatment
- Too time-consuming to learn how to complete new forms

**FACILITATOR TO GETTING STARTED**

- What is needed to start CPP treatment?

Lastly, is there anything we haven’t discussed that you think would be good for us to know?

Thank you very much for participating today
