# Supplementary material for: Determinants of implementation of child-parent psychotherapy to treat early childhood trauma: a reflexive analysis
Source: BMC Health Serv Res. 2025 Jul 3;25:907. doi: 10.1186/s12913-025-12937-w (PMC12224486; doi:10.1186/s12913-025-12937-w)
Supplement: Supplementary file 2 — Supplementary Material 2. [file 12913_2025_12937_MOESM2_ESM.docx]

**Appendix B**

**Interview guide follow-up**

**ACCEPTANCE AND ADAPTATION**

- How does this way of working function in practice?
  - Do you like working this way?
  - What works less well? What practical challenges are there?
- Do you find that CPP meets the needs of children and parents exposed to violence or trauma?
  - Is there a need for CPP in your practice?
  - In what way(s) is CPP better or worse than other interventions for a similar target group?
- How do parents and children respond to the method?
- Is there anything in the method that you feel skeptical about?
  - Are there difficult or unnecessary elements/aspects?
- Do you have specific suggestions for how CPP can be adapted (to a Norwegian setting/your workplace)?

**FEASIBILITY**

- Have there been situations/cases where it was not appropriate to work with this method?
  - Examples?
- On what basis have parents declined this method?

**CONTINUATION**

- Will you continue using this method when the training is completed? (ask participants to raise their hands and count aloud)
  - Why or why not?
  - Can you foresee obstacles/barriers for continuing work with the method?
- Would you like more therapists at your workplace to learn the method?
  - Will they have the opportunity to do so?
- Do you have genuine support from your management to continue working with this method?
  - In what way does management facilitate continued work with CPP?
  - Does management encourage working with CPP?
  - Do you receive recognition for the work you put into CPP? (in what way?)
